# Association between virtues and posttraumatic growth: preliminary evidence from a Chinese community sample after earthquakes

**DOI:** 10.7717/peerj.883

**Published:** 2015-04-07

**Authors:** Wenjie Duan, Pengfei Guo

**Affiliations:** 1 Department of Applied Social Sciences, City University of Hong Kong, Hong Kong, China; 2 Hospital (T. C. M) Affiliated to Luzhou Medical College, Luzhou, Sichuan, China

**Keywords:** Post-traumatic growth, Trauma, Virtues, Relationship, Conscientiousness, Vitality

## Abstract

**Objective.** Relationship, vitality, and conscientiousness are three fundamental virtues that have been recently identified as important individual differences to health, well being, and positive development. This cross-sectional study attempted to explore the relationship between the three constructs and post-traumatic growth (PTG) in three directions, including indirect trauma samples without post-traumatic stress disorder (PTSD), direct trauma samples without PTSD, and direct trauma samples with PTSD.

**Methods.** A total of 340 community participants from Sichuan Province, Mainland China involved in the study, most of which experienced Wenchuan and Lushan Earthquake. Participants were required to complete the self-reported questionnaire packages at one time point for obtaining their scores on virtues (Chinese Virtues Questionnaire), PTSD (PTSD Checklist-Specific), and PTG (Post-traumatic Growth Inventory-Chinese).

**Results.** Significant and positive correlations between the three virtues and PTG were identified (*r* = .39–.56; *p* < .01). Further regression analysis by stepwise method reveled that: in the indirect trauma samples, vitality explained 32% variance of PTG. In reference to the direct trauma sample without PTSD, both relationship and conscientiousness explained 32% variance of PTG, whereas in the direct trauma sample with PTSD, only conscientiousness accounted for 31% the variance in PTG.

**Conclusion.**This cross-sectional investigation partly revealed the roles of different virtues in trauma context. Findings suggest important implications for strengths-based treatment.

## Introduction

Natural disasters, cancer, bereavement, and other life-threatening events with potentially irreversible consequences often lead to positive changes and transcendence known as posttraumatic growth (PTG, Calhoun & Tedeschi, 2006; Tedeschi & Calhoun, 2004). A promising research direction focuses on the effects of personal strengths on coping and responses to health- and wellbeing-related concerns and worries, such as traumatic events (Hampson & Friedman, 2008).

To date, the most systematic approach for studying personal strengths is the Values in Action Classification developed by Peterson & Seligman (2004); this approach included 24 character strengths (e.g., hope, self-regulation, and gratitude) grouped into six core virtues (e.g., wisdom, courage, and humanity). They conceptualized virtue as “a property of the whole person and the life that person leads” (p. 87), which is a personal strength appreciated by the whole society (Peterson & Seligman, 2004). In the past decade, many studies revealed the positive relationship between these positive qualities and mental health (for review, see Niemiec, 2013) and demonstrated that the use of strengths is a valid approach for enhancing wellbeing in diverse populations (Duan et al., 2014; Seligman et al., 2005). Accordingly, the relationship between these qualities and PTG was explored using 1,739 samples from different countries (Peterson et al., 2008). Findings indicated that kindness, love, bravery, hope, and religiousness show stronger relations with PTG than other strengths (Peterson et al., 2008). However, no correlation coefficient was higher than 0.35, thus reflecting weak correlation. Principal component factor analysis revealed a five-factor structure of virtues, namely, interpersonal, fortitude, cognitive, transcendence, and temperance. Correlation analysis showed that all these five virtues correlated with PTG, but all the correlation coefficients were lower than 0.21 (Peterson et al., 2008).

Three issues should be noted regarding the study of Peterson et al. (2008). First, the virtue structure in the aforementioned study is questionable. Various studies have found that the 24 identified strengths can be grouped into different virtues in diverse cultures, and the groups include 3-, 4-, or 5-factor structures (Duan et al., 2012b; Ho, Duan & Tang, 2014a). Thus, the virtue structure should be clarified prior to delineating the function of virtues in facing trauma and should be explored and analyzed further. Duan et al. (2012b) adopted the combined emic and etic approaches to select 96 cross-culturally equivalent items from the original 240-item Values In Action Inventory of Strengths by using a Chinese population (Ho et al., 2014b). Exploratory and confirmatory factor analyses revealed 3 virtues, namely, relationship, vitality, and conscientiousness (Duan et al., 2013; Zhang et al., 2014a). The relationship virtue reflects “the love, concern, and gratitude of a person toward others”; vitality reflects “the curiosity and zest for creativity of an individual”; and conscientiousness is “an intrapersonal virtue that describes people who persist in achieving goals and exhibit self-control,” which reflects the individual orientation of the virtues (Ho, Duan & Tang, 2014a). A recent study conducted by McGrath (2014) likewise demonstrated the three-virtue structure by investigating multiple inventories and large samples. Therefore, the relationship between the three virtues and PTG should be reexamined. Second, the research conducted by Peterson et al. (2008) examined only the relationship among trauma samples, which accounted for 56% of the entire population. However, previous studies argued that stress-related life events could likewise facilitate stress-related growth, which is assessed by Posttraumatic Growth Inventory (PTGI), but to a lesser extent than traumatic events (e.g., LoSavio et al., 2011). The level of perceived stress that resulted from events might be the key cause of PTG, rather than the objective and specific events per se. Third, several individuals who underwent trauma may develop posttraumatic stress disorder (PTSD), whereas others may not (Yehuda & Flory, 2007). The above study failed to consider the influence of PTSD on PTG in the trauma samples. An inverted-U curve was also found between PTG and PTSD, which suggested that PTG decreased after a moderate level of PTSD (Levine et al., 2008). Nevertheless, an increasing interest was noted among mental health professionals in determining the strengths of their clients (McCrae, 2011).

## Current Study

To expand our understanding of the function of strengths in PTG, the relationship between virtues and PTG should be explored. Basing on the above literature review, PTG in the current study refers to growth following stress-related events, including daily stressors and traumatic events. Individuals indirectly exposed to traumatic events can be recognized as persons who experienced stress-related events. Accordingly, both direct and indirect trauma groups would acquire PTG, and the differences between these two groups would be insignificant (Hypothesis 1). As previously discussed, a few traumatic individuals may develop PTSD, which implied that the contributions of the three virtues (relationship, vitality, and conscientiousness) to PTG vary depending on trauma type (i.e., direct trauma vs. indirect trauma) and PTSD status (i.e., PTSD group vs. non-PTSD group) (Hypothesis 2). The current results will clarify the contributions of virtues in traumatic situations, which may facilitate a strength-based approach in both research and practice in the future.

## Method

### Participants

A total of 340 qualified respondents (109 males and 231 females) were recruited from different communities in Dujiangyan area, Sichuan, China, which were affected by the 2008 earthquake. A total of 69 participants were aged 18–25, 101 were in the range of 36–35, 112 were in the range of 36–45, and 58 were above 46 years old. Only 51 participants have obtained a university degree or above. As expected, “natural disaster,” “sudden or unexpected death of someone close to you,” and “life-threatening illness or injury” were the three most endorsed items listed on the questionnaire on traumatic events (Table 1).

**Table 1:** Demographic and sample characteristics.

Variables	*n*	%
Gender		
Male	109	32.06%
Female	231	67.94%
Age		
18–25	69	20.29%
26–35	101	29.71%
36–45	112	32.94%
46 and above	58	17.06%
Education		
Secondary school and below	37	10.88%
Tertiary school	186	54.71%
College	66	19.41%
University and above	51	15.00%
Types of trauma		
Natural disaster	126	37.06%
Sudden, unexpected death of someone close to you	102	30.00%
Life threatening illness or injury	56	16.47%
Fire or explosion	49	14.41%
Transportation accident	33	9.71%

### Procedures

The announcement for study participation was published on community bulletin boards, which can be seen by most people who lived in the community. Individuals who were interested to participate were instructed to complete first the Life Events Checklist (LEC), and only the participants who directly or indirectly experienced trauma were qualified to complete the other scale during the following week (screening criteria are described in the ‘Measures’ section). The Human Subjects Committee of Traditional Chinese Medicine Hospital Affiliated to Luzhou Medical College approved the study. All data collected were anonymous and confidential. Psychological assistance was provided to protect the subjects. Data were collected from December 2013 to April 2014.

### Measures

#### Life Events Checklist

LEC was used to screen individuals who experienced direct or indirect trauma through 17 potential events (Gray et al., 2004). Participants are requested to rate each event on a five-point Likert scale (1 = happened to me, 2 = witnessed it, 3 = learned about it, 4 = not sure, 5 = does not apply). Participants who indicated at least one traumatic event as 1 = “happened to me” were defined as direct trauma samples, whereas respondents who indicated 2 = “witnessed it” and/or 3 = “learned about it” were indirect trauma samples. Participants who selected 4 = “not sure” and/or 5 “does not apply” in the checklist were excluded. Considering that all participants involved in this study were sampled within the earthquake zone (Dujiangyan area in Sichuan Province), rather than other place far away from the earthquake-prone area, they should not be treated as persons who lived far away from Sichuan and were affected by the earthquake only through radio and television. Thus, all the qualified participants may have experienced at least some indirect exposure to earthquake.

#### Chinese Virtues Questionnaire

Virtues were assessed using the Chinese Virtues Questionnaire, which is a 96-item simplified Chinese scale with good psychometric characteristics (Duan et al., 2013; Duan et al., 2012b). The respondents were requested to rate each item from 1 (very much unlike me) to 5 (very much like me) on a five-point Likert scale. Item samples include “I can accept love from others” (Relationship), “I like to think of new ways to do things” (Vitality), and “I control my emotions” (Conscientiousness). A high mean score reflects a high degree of the virtue within an individual. In this study, the Cronbach’s *α* values of the three subscales were 0.91 (relationship), 0.85 (vitality), and 0.84 (conscientiousness).

#### Posttraumatic Growth Inventory-Chinese.

A 15-item Chinese version of the PTGI (Ho et al., 2004) measures growth following a traumatic event. The measurement requires individuals to indicate the extent of their experiences of changes as a result of crisis, ranging from 0 (not at all) to 5 (a very great degree). The reliability and validity of the 15-item version were accurate in previous studies (Ho et al., 2004). In the present sample, the Cronbach’s *α* of the inventory was 0.84.

#### PCL-S

PTSD symptoms were evaluated by the 17-item PCL-S. Participants are requested to rate their experience from 1 (not at all) to 5 (extremely). Previous studies demonstrated that the Chinese version can be used as a screening questionnaire among the Chinese population (Li et al., 2010). Scores of 44 or above indicate a PTSD diagnosis (Blanchard et al., 1996). Responses of the diagnosed PTSD participants also fulfilled the criterions of DSM-IV, including (a) “history of a traumatic stressor,” (b) “persistent re-experiencing of the traumatic event,” (c) “persistent avoidance of stimuli associated with the trauma and numbing of general responsiveness,” and (d) “persistent symptoms of increased arousal.” In the current sample, the Cronbach’s *α* of the entire scale was 0.93.

## Results

The descriptive statistics of all variables are listed in Table 2. ANOVA showed that the virtue of relationship and PTG exhibited significant differences among the three samples (*p* < 0.05). Post-hoc tests further revealed that both the relationship and PTG of direct trauma with PTSD sample were significantly lower than those of the other groups (*p* < 0.05). Correlation analysis (Table 3) revealed that different virtues showed significantly positive relations with PTG in the total sample and three subsamples, ranging from 0.39 to 0.56 (*p* < 0.01).

**Table 2:** Descriptive statistics and group differences.

	Indirect trauma sample (*n* = 88)		Direct trauma without PTSD Sample (*n* = 217)		Direct trauma with PTSD Sample (*n* = 35)		ANOVA	
	*M*	*SD*	*M*	*SD*	*M*	*SD*	*F*	*Sig.*
Relationship	4.24	.41	4.17	.52	3.93	.51	5.08	.01
Vitality	4.16	.47	4.16	.44	4.02	.37	1.58	.21
Conscientiousness	4.01	.49	3.87	.56	3.89	.49	2.30	.10
PTG	3.34	.62	3.34	.56	3.09	.41	3.11	.05
PTSD	–	–	2.01	.39	3.26	.41	–	–

**Table 3:** Correlations between virtues and posttraumatic growth in different subgroups.

	Posttraumatic growth			
	Total sample	Indirect trauma sample (*n* = 88)	Direct trauma without PTSD sample (*n* = 217)	Direct trauma with PTSD sample (*n* = 35)
Relationship	.48^**^	.44^**^	.50^**^	.53^**^
Vitality	.54^**^	.56^**^	.39^**^	.45^**^
Conscientiousness	.46^**^	.49^**^	.45^**^	.56^**^

**Notes.**

** *p* < .01.

Further regression analysis by stepwise method revealed that virtues served different functions in diverse subsamples (Table 4). In the indirect trauma sample, only vitality (*Beta* = 0.56, *t* = 6.44, *p* < 0.001) significantly explained 32% variance of PTG, whereas based on the direct trauma without PTSD sample, relationship (*Beta* = 0.38, *t* = 5.98, *p* < 0.001) and conscientiousness (*Beta* = 0.29, *t* = 4.54, *p* < 0.001) also accounted for 32% variance of PTG. Finally, in the direct trauma with PTSD sample, only conscientiousness (*Beta* = 0.56, *t* = 3.85, *p* < 0.01) can contribute 31% explained variance to PTG.

**Table 4:** Regression of virtues on posttraumatic growth in different subgroups.

	Indirect trauma sample (*n* = 88)				Direct trauma without PTSD sample (*n* = 217)				Direct trauma with PTSD sample (*n* = 35)			
	*R* ^2^	*F*	*Beta*	*t*	*R* ^2^	*F*	*Beta*	*t*	*R* ^2^	*F*	*Beta*	*t*
	.32	40.05^***^			.32	50.22^***^			.31	14.827^**^		
Relationship			.07	.53			.38	5.98^***^			.27	1.29
Vitality			.56	6.44^***^			.07	1.04			.23	1.36
Conscientiousness			.17	1.37			.29	4.54^***^			.56	3.85^**^

**Notes.**

** *p* < .01.

*** *p* < .001.

## Discussion

This research is based on a community sample of people directly or indirectly exposed to trauma (e.g., earthquakes). As expected, no difference of PTG existed between the indirect and direct trauma samples. A previous study indicated that a population indirectly hit by an earthquake could still grow after traumatic experiences (Yu et al., 2010). In the present study, some participants directly experienced an earthquake, whereas some might have indirectly experienced this event through their friends, witnessed the death of a close friend, or experienced the psychological distress caused by the death of a family member or a close friend. Thus, all the participants may undergo PTG. The growth also decreased with increased severity of PTSD, which was partly explained by the inverted-U curve between PTG and PTSD. As reflected by the present results, the correlation between PTG and PTSD was almost significant and negative, particularly in the PTSD sample (*r* = − 0.31, *p* = 0.07). Mol et al. (2005) also investigated 832 individuals and found that the scores of their PTSD were the same for some early-life and traumatic events, which partly revealed that some life events could also generate PTSD symptoms. All these results suggest that the objective traumatic events may not essentially lead to PTG or PTSD. According to the Transactional Model of Stress and Coping (Lazarus & Folkman, 1984), the perceived stress from these events could be triggered. Thus, further studies are necessary to understand the differences in PTG and PTSD after traumatic and non-traumatic events.

Our previous studies preliminarily demonstrated that three virtues are positively associated with positive health outcomes (Duan et al., 2012a; Duan et al., 2013; Tang et al., 2014), such as satisfaction with life and flourishing, but negatively associated with negative health outcomes (Duan et al., 2013; Duan et al., 2015; Zhang et al., 2014c), such as depression, anxiety, general severity index, and pathological Internet use. These results indicated that the three virtues might be protective factors of mental health. Accordingly, personal virtues may facilitate rebounding for individuals who experienced trauma.

Individuals with different endorsed virtues often occupy different psychological resources (e.g., optimism, emotional control, and gratitude). Thus, they can maximize the use of different resources (i.e., diverse virtues) in various contexts. For instance, an individual with a high level of relationship virtue is more adept at obtaining social support from his/her friends and relatives to recover from the trauma and even obtain growth. Thus, the main results of the current study revealed the different potential functions of virtues (i.e., relationship, vitality, and conscientiousness) in PTG. In the indirect trauma samples, only vitality contributed to the variance of growth after trauma. In the direct trauma without PTSD sample, relationship and conscientiousness explained the variance of PTG, whereas in the PTSD sample, only conscientiousness was the significant contributor. Basing on the previous and current findings, we can speculate the different functions of virtues in various samples. Without directly experiencing trauma, most people are troubled by small stress, and some are indirectly affected by trauma. When similar scenarios occur, vitality can cause individuals to perceive less stress, thereby reducing psychological distress (Duan et al., 2015). Our previous study identified that only students with higher vitality have perceived less stress from minor life events, which consequently introduced less psychological distress (Duan et al., 2015). Individuals with high vitality are also more willing to express their concerns to relieve stress and improve mental health (Yang et al., 2015; Zhang et al., 2014b). After being directly exposed to traumatic events, most people retain psychological balance without significant symptoms of PTSD (Bonanno et al., 2007). Therefore, interpersonal resources related to the virtue of relationship are necessary. Prati & Pietrantoni (2009) demonstrated that social support is a significant contributor to PTG in a meta-analysis of 103 studies. Individuals who are rated high in the virtue of relationship are more likely to adopt a supportive mechanism to overcome the predicaments caused by trauma. Finally, conscientiousness, also termed as self-regulation, always facilitates positive mental health and decreases psychopathology (Hagger, 2010). Duan et al. (2015) found that individual conscientiousness can directly decrease psychological distress, regardless of the level of stress. Consequently, the intrapersonal strengths reflected by conscientiousness can be used to regulate emotion, cognition, and behavior to resolve conflicts caused by trauma, thus enhancing growth after trauma.

In fact, the three virtues (i.e., relationship, vitality, and conscientiousness) researched here correspond strongly to the three well-established character traits proposed by Cloninger & Zohar (2011), namely cooperativeness, which is consistent with relationship; self-directedness, which is consistent with vitality; and self-transcendence, which is consistent with conscientiousness. These character traits and the three virtues, according to the Cloninger (2004)’s psychobiological theory of personality, were cognitive domain of personalities, which were more stable and developed across lifespan toward social norms that approved and appreciated by the whole society (Josefsson et al., 2013b). Previous studies found that high self-directedness as a health-promoting trait that associated with coping with prior trauma (North, Abbacchi & Cloninger, 2012). Other longitudinal studies revealed the predictive ability of character traits to well being (Cloninger & Zohar, 2011; Josefsson et al., 2013a). These findings and abovementioned studies on virtues suggest that both character traits and virtues may be helpful in coping with challenges and enhancing well-being. For instance, Eley et al. (2013) recently revealed the associations between profile of temperament & character and resilience, indicating the importance of high levels of self-directedness, cooperativeness, and persistence. Our ongoing cross-sectional study likewise preliminarily found that virtues and trait resilience were conceptually related (Duan, Guo & Gan, 2015). However, future longitudinal studies should be operated to explore the predictive ability of virtues in traumatic context and further reveal the possible causal relationship between virtues and resilience.

Some limitations should be noted. First, it should be stressed again that, similar to some studies on trauma, the design of this study was cross-sectional rather than longitudinal. Results of this research and the hypothesized functions of virtues in the development process of a traumatic event should be replicated and examined in future longitudinal designs. Second, the direct and indirect trauma samples were divided based on self-reported measurements, wherein “indirect trauma” could be anything from hearing about the traumatic event from a friend to watching it on TV, although they lived in the earthquake zone. These participants were actually not trauma-exposure types according to DSM or ICD, thereby leaving future studies to distinguish the two samples objectively. Third, all the data were collected by self-reported method, which may introduce common method bias. Future studies should adopt multiple methods for controlling the bias. Finally, this study is the first to examine the function of virtues in trauma research among Chinese. More psychological outcomes, including flourishing, depression, and anxiety, should be explored in the future.

## Supplemental Information

10.7717/peerj.883/supp-1 Supplemental Information 1 Dataset
